# Diabetes, Hypertension, and Cardiovascular Disease: Clinical Insights and Vascular Mechanisms

**DOI:** 10.1016/j.cjca.2017.12.005

**Published:** 2018-05

**Authors:** John R. Petrie, Tomasz J. Guzik, Rhian M. Touyz

**Affiliations:** Institute of Cardiovascular and Medical Sciences, University of Glasgow, United Kingdom

## Abstract

Hypertension and type 2 diabetes are common comorbidities. Hypertension is twice as frequent in patients with diabetes compared with those who do not have diabetes. Moreover, patients with hypertension often exhibit insulin resistance and are at greater risk of diabetes developing than are normotensive individuals. The major cause of morbidity and mortality in diabetes is cardiovascular disease, which is exacerbated by hypertension. Accordingly, diabetes and hypertension are closely interlinked because of similar risk factors, such as endothelial dysfunction, vascular inflammation, arterial remodelling, atherosclerosis, dyslipidemia, and obesity. There is also substantial overlap in the cardiovascular complications of diabetes and hypertension related primarily to microvascular and macrovascular disease. Common mechanisms, such as upregulation of the renin-angiotensin-aldosterone system, oxidative stress, inflammation, and activation of the immune system likely contribute to the close relationship between diabetes and hypertension. In this article we discuss diabetes and hypertension as comorbidities and discuss the pathophysiological features of vascular complications associated with these conditions. We also highlight some vascular mechanisms that predispose to both conditions, focusing on advanced glycation end products, oxidative stress, inflammation, the immune system, and microRNAs. Finally, we provide some insights into current therapies targeting diabetes and cardiovascular complications and introduce some new agents that may have vasoprotective therapeutic potential in diabetes.

## Résumé

L’hypertension et le diabète de type 2 sont des affections concomitantes fréquentes. L’hypertension est deux fois plus fréquente chez les patients atteints de diabète que chez ceux qui n’en sont pas atteints. De plus, les patients atteints d’hypertension sont souvent résistants à l’insuline et sont plus susceptibles de souffrir de diabète que les personnes normotendues. Chez les diabétiques, la principale cause de morbidité et de mortalité est la maladie cardiovasculaire, qui est exacerbée par l’hypertension. En conséquence, le diabète et l’hypertension sont étroitement interreliés en raison de facteurs de risques similaires, comme la dysfonction endothéliale, l’inflammation vasculaire, le remodelage artériel, l’athérosclérose, la dyslipidémie et l’obésité. On observe un chevauchement important entre les complications cardiovasculaires du diabète et celles de l’hypertension liées principalement à des maladies microvasculaires et macrovasculaires. Des mécanismes communs, comme une stimulation du système rénine-angiotensine-aldostérone, un stress oxydatif, une inflammation et une activation du système immunitaire, sont susceptibles de contribuer à la relation étroite entre le diabète et l’hypertension. Dans cet article, nous abordons le diabète et l’hypertension comme des affections concomitantes et nous parlons des caractéristiques physiopathologiques des complications vasculaires associées à ces affections. Nous soulignons également certains mécanismes vasculaires qui prédisposent à ces deux affections, en mettant l’accent sur les produits finaux de glycation avancée, le stress oxydatif, l’inflammation, le système immunitaire et les micro-ARN. Finalement, nous présentons certaines connaissances sur les traitements actuels ciblant le diabète et les complications cardiovasculaires et nous présentons de nouveaux agents qui pourraient avoir un pouvoir vasoprotecteur chez les patients diabétiques.

## Type 2 Diabetes Mellitus and Hypertension

The prevalence of obesity and type 2 diabetes (T2D) continues to rise worldwide as lifestyles associated with low energy expenditure and high caloric intake are increasingly adopted, particularly in lower-income and developing countries. It is predicted that the number of cases of T2D will rise from 415 million to 642 million by 2040.^1^ Hypertension is even more common, rising in prevalence in the same countries, with a recent worldwide estimate of 1.39 billion cases.^2^

Although T2D and hypertension can be simply diagnosed at the bedside, they are each complex and heterogeneous phenotypes associated with an elevated risk of life-threatening cardiovascular disease (CVD). Their frequent coexistence in the same individual is not a coincidence, because aspects of the pathophysiology are shared by both conditions, particularly those related to obesity and insulin resistance. For example, in the San Antonio Heart Study, 85% of those with T2D had hypertension by the fifth decade of life, whereas 50% of those with hypertension experienced impaired glucose tolerance or T2D.^3^

In health, insulin maintains glucose homeostasis by integrated actions on carbohydrate, protein, and lipid metabolism. Loss of sensitivity to aspects of insulin action (insulin resistance) principally affects the liver, muscle, and adipose tissues and is selective for glucose and lipid metabolism, eg, sparing insulin's action to retain sodium in the distal tubule.^4^, ^5^ Reduction in insulin-mediated glucose disposal leads to compensatory hypersecretion of insulin to maintain homeostasis: Glucose intolerance ensues if this endocrine pancreas response is inadequate, although some obese individuals avoid T2D by virtue of a supranormal B-cell response.^6^ Recently, the role of adipose tissue in these associations has been increasingly appreciated.^7^

**Figure 1:**
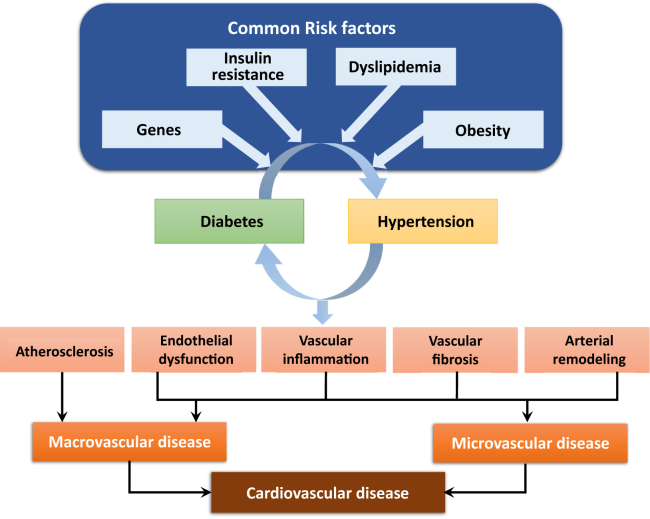
Vascular processes whereby diabetes and hypertension predispose to cardiovascular disease. Common risk factors promote diabetes and hypertension, which are associated with atherosclerosis, vascular inflammation, endothelial dysfunction, and structural remodelling, which lead to macrovascular and microvascular disease. Vascular damage and endothelial dysfunction is amplified when diabetes and hypertension coexist.

Diabetes is associated with both macrovascular (involving large arteries such as conduit vessels) and microvascular (involving small arteries and capillaries) disease. Chronic hyperglycemia and insulin resistance play an important role in the initiation of vascular complications of diabetes and involve a number of mechanisms including (1) increased formation of advanced glycation end products (AGEs) and activation of the receptor for advanced glycation end products (RAGE) AGE-RAGE axis, (2) oxidative stress, and (3) inflammation.^8^ In addition, emerging evidence suggests a role for microRNAs (miRNAs) in the vasculopathy of diabetes (see further on).^9^ Hypertension is an important risk factor for diabetes-associated vascular complications, because hypertension itself is characterized by vascular dysfunction and injury (Fig. 1).

In this review, we focus on vascular complications of diabetes and discuss the impact of comorbidities, specifically hypertension. The role of oxidative stress and inflammation as “common soil” for metabolic and vascular disease are highlighted. We also discuss how some of the newer agents used in the treatment of T2D can influence blood pressure (BP) regulation and the risk of CVD, with an eye to future developments more specifically targeting vascular protection.

## Macrovascular Disease

### Clinical features

Macrovascular (or cardiovascular) disease of larger conduit arteries is a complex inflammatory process leading to myocardial infarction, stroke, and peripheral artery disease. The primary pathologic process associated with macrovascular disease is atherosclerosis, which in diabetes is accelerated with extensive distribution of vascular lesions.^10^ T2D confers an approximate 2-fold elevation in CVD risk, equivalent to that of a previous myocardial infarction.^11^, ^12^ Moreover, patients with T2D have poorer outcomes after an acute coronary syndrome and higher rates of reinfarction and heart failure.^13^

Elevation of CVD risk begins at the stage of prediabetes in association with insulin resistance and impaired glucose tolerance.^14^ As well as being the diagnostic hallmark of T2D, hyperglycemia is the principal determinant of microvascular complications of T2D and plays an important role in the pathogenesis of CVD. However, in established T2D, it is a relatively weak modifiable risk factor compared with hypertension, dyslipidemia, and (unfortunately in many populations) cigarette smoking.^15^, ^16^

### Pathophysiological features

Insulin resistance is detectable for several years before the onset of T2D. It is associated with obesity, particularly central obesity, but may be present in lean individuals with hypertension.^17^ During calorie excess, adipocytes in obese humans—whether in subcutaneous or visceral areas—undergo hypertrophy. Visceral adipocytes are more susceptible to cellular death as they begin to enlarge and their stromal vascular fraction becomes infiltrated with macrophages.^18^

These macrophages around dead adipocytes form “crown-like structures,” a histologic appearance that is associated with expression of cytokines (including tumor necrosis factor-α [TNF-α], interleukin-6 [IL-6]), and inducible nitric oxide synthase.^19^ These changes have been shown to coincide with the onset of insulin resistance and provide a pathophysiological link between metabolic and vascular disease.^20^

In addition to these proinflammatory changes, adipocyte hypertrophy is associated with larger triglyceride stores, a higher lipolytic rate, and an atherogenic lipid profile: elevated concentrations of small dense low-density lipoprotein cholesterol, high concentrations of triglycerides, triglyceride-rich remnants, very low-density lipoprotein cholesterol, and apolipoprotein B, usually in combination with low levels of high-density lipoprotein cholesterol.^7^ This profile is associated with increased production of leptin, decreased production of adiponectin, higher circulating levels of nonesterified fatty acids (NEFAs), and activation of mitochondrial oxidative stress pathways in vascular endothelial cells.^7^

These proinflammatory and metabolic consequences of obesity and insulin resistance result in endothelial dysfunction, a key antecedent and modulator of atherosclerosis that has been demonstrated not only in hypertension but also in prediabetes,^21^ first-degree relatives of individuals with T2D,^22^ and even insulin-resistant healthy individuals.^22^, ^23^ It is characterized by disruption of the intricate physiological balance between vasoconstrictors (endothelin, angiotensin II) and vasodilators (nitric oxide, prostacyclin), growth promoting and inhibitory factors, proatherogenic and antiatherogenic factors, and procoagulant and anticoagulant factors.^24^, ^25^ A substantial body of evidence suggests that impaired endothelium-dependent vasodilation may in turn contribute to or exacerbate insulin resistance by limiting the delivery of substrate (glucose) to key target tissues.^26^

In addition to these functional changes, an associated low-grade inflammation in endothelial and smooth muscle cells of the vascular wall causes cell proliferation, hypertrophy, remodelling, and apoptosis.^27^ This accelerates disruption of the balance between the arterial wall scaffolding proteins elastin and collagen that determine vascular compliance, a form of “vascular aging,” which is a characteristic phenotype in hypertension.^28^, ^29^, ^30^, ^31^ Vascular stiffening leads to widening of arterial pulse pressure and increased pulsatile shear, exacerbating endothelial dysfunction and vascular disease.^32^

## Microvascular Disease

### Clinical features

Microvascular disease leads to retinopathy, nephropathy, and neuropathy, which are major causes of morbidity and mortality in patients with diabetes. In the United States, diabetic retinopathy affects about 28% of individuals with established T2D.^33^ Worldwide it is responsible for 10,000 cases of blindness every year.^34^, ^35^ Diabetic nephropathy affects about 25% of individuals with T2D and is the most common cause of renal failure in the United States.^36^ Neuropathy affects about 20% of these individuals, although it is estimated that about 50% have neuropathy at some point in their lives.^36^ Each of these organ-specific microvascular complications has its own unique clinical and histologic features, but all are common with increasing duration of hyperglycemia and are driven by its downstream cellular effects, including polyol accumulation (resulting from saturation of the hexokinase pathway and consequent increased activity of aldose reductase), AGE-induced injury, increased vascular permeability, and oxidative stress.^8^

Follow-up of the **A**ction in **D**iabetes and **Va**scular Disease: Preterax and Diamicro**n C**ontrolled **E**valuation (ADVANCE) trial cohort has confirmed that the presence of microvascular complications increases the risk of cardiovascular complications in individuls with T2D.^37^ Moreover, the coexistence of hypertension and retinopathy is a risk factor for the progression of nephropathy. There is evidence that treatment of hypertension with angiotensin II receptor blockers can reduce the progression of retinopathy in addition to well-known effects on nephropathy.^38^

### Pathophysiological features

Pathognomonic alterations of diabetic microangiopathy include capillary basement membrane thickening, increased endothelial permeability, and endothelial and vascular smooth muscle cell dysfunction. Hyperglycemia is the key stimulus for these processes by stimulating vasoinjurious signalling pathways, activating the polyol pathway, increasing oxidative stress, stimulating proinflammatory transcription factors, and activation of immune responses. Similar processes are induced by hypertension.^39^

## Mechanisms of Vascular Complications in Diabetes and the Impact of Hypertension

**Figure 2:**
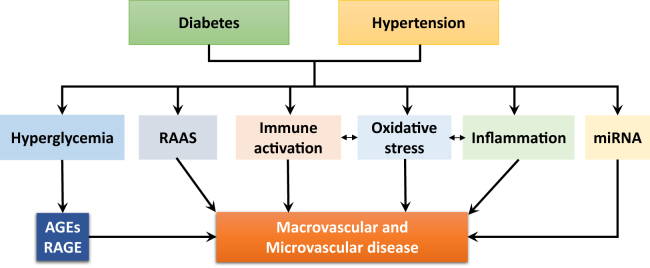
Putative mechanisms whereby diabetes and hypertension cause vascular disease. Immune cell activation and inflammation are mediated through oxidative stress. AGEs, advanced glycation end products; RAAS, renin-angiotensin-aldosterone system; RAGE, receptor AGE.

A number of interacting mechanisms are in play as summarized in the following sections (Fig. 2).

### AGE-RAGE axis

AGEs are compounds that have undergone irreversible posttranslational modifications because of reactions between sugars and amino groups on proteins and nucleic acids. Hyperglycemia accelerates formation of AGEs, which accumulate in the extracellular matrix of vessels and contribute to vascular damage in diabetes.^40^ AGEs stimulate production of reactive oxygen species (ROS), which in turn further enhance AGE formation. AGEs are also antigenic and hence induce immune responses.^40^ In addition to AGEs, dicarbonyl methylglyoxal, a by-product of glycolysis, accumulates in tissues and contributes to diabetes-associated vascular damage.^41^

AGEs interact with 2 main types of cell surface receptors: (1) scavenger receptors, which remove and degrade AGEs, and (2) receptors for AGEs (RAGE), which trigger specific cellular signalling responses on AGE binding. RAGE is a member of the immunoglobulin family and binds many ligands besides AGEs, such as high mobility group protein B1, S100 calcium-binding proteins (including calgranulin), amyloid-β-protein, and amphotericin. AGE-RAGE signals through transforming growth factor (TGF)-β, NF-κB, mitogen-activated protein kinases (MAPK; ERK1/2, p38MAPK), and nicotinamide adenine dinucleotide phosphate (NADPH) oxidases (Nox) and induces expression of vascular adhesion molecule 1, E-selectin, vascular endothelial growth factor, and proinflammatory cytokines (IL-1β, IL-6, TNF-α).^42^ In diabetes, activation of these signalling pathways is increased in vascular smooth muscle cells, leading to vascular fibrosis, calcification, inflammation, prothrombotic effects, and vascular damage, processes underlying diabetic nephropathy, retinopathy, neuropathy, and atherosclerotic CVD.^43^ Coexisting hypertension amplifies these complications and contributes to the accelerated vasculopathy in diabetes.^44^ Patients with diabetes have increased tissue and circulating concentrations of AGEs and soluble RAGE, which is predictive of cardiovascular events and all-cause mortality. As such, urinary and plasma AGE levels and soluble RAGE may act as biomarkers for vascular disease in diabetes.^45^

Targeting AGE-RAGE has been considered a potential therapeutic strategy to reduce or prevent CVD in diabetes. A number of large clinical trials investigating cardiovascular benefits of alagebrium (ALT-711), which reduces accumulation of AGEs by cleaving AGE cross-links, have been undertaken. They include Distensibility Improvement and Remodeling in Diastolic Heart Failure **(**DIAMOND; NCT00043836), Systolic and Pulse Pressure Hemodynamic Improvement By Restoring Elasticity (SAPPHIRE; NCT00045981), **S**ystolic Hypertension **I**nteraction With **L**eft **Ve**ntricular **R**emodeling (SILVER; NCT00045994), **S**ystolic **P**ressure **E**ffica**c**y and Safety **Tr**ial of **A**lagebrium (SPECTRA; NCT00089713), **B**eginning a **R**andomized **E**valuation of the **A**GE Brea**k**er Alagebrium in **D**iastolic **H**eart **F**ailure I (BREAK-DHF-I; NCT00662116), and Evaluating the Efficacy and Safety of Alagebrium (ALT-711) in Patients With Chronic Heart Failure (BENEFICIAL; NCT00516646). However few data have been published from these studies. Some small clinical studies demonstrated cardiovascular benefit in patients with diabetes and hypertension.^46^ In particular, alagebrium improved endothelial function, reduced aortic stiffness, and increased vascular compliance.^47^

### Oxidative stress and Nox

Oxidative stress is a key mechanism of glucotoxicity in diabetes, as evidenced by increased vascular ROS generation in response to hyperglycemia and accumulation of oxidation by-products of lipids, proteins, and nucleic acids.^27^ NADPH oxidases and dysfunctional endothelial nitric oxide synthase are principal sources of increased ROS in human vasculature in T2D.^48^, ^49^ ROS interact with DNA and stimulate many redox-sensitive signalling pathways that lead to inflammation, fibrosis, and vascular damage. Increased vascular oxidative stress in diabetes and hypertension promotes posttranslational oxidative modification of proteins, causing cellular damage and vascular dysfunction. Hyperglycemia also induces activation of redox-sensitive protein kinase C and polyol and hexosamine pathways, further contributing to mitochondrial dysfunction, oxidative stress, endoplasmic reticulum stress, and consequent cellular damage.^50^ Oxidative stress is also associated with reduced bioavailability of the vasodilator nitric oxide, causing endothelial dysfunction.

Diabetes-induced oxidative stress is caused by numerous processes, including glucose-stimulated mitochondrial respiration, endoplasmic reticulum stress, activation of the renin-angiotensin system (which is pro-oxidant), decreased vascular antioxidant capacity, reduced activity of the master antioxidant transcription factor nuclear factor-erythroid 2-related factor (Nrf-2), and activation of Nox isoforms.^51^ Of these mechanisms activation of Nox types is particularly important. Four Nox isoforms have been demonstrated in human vessels, including Nox1, Nox2, Nox4, and Nox5. Nox-derived ROS influence redox-sensitive signalling pathways in vascular cells such as MAPKs, protein tyrosine phosphatases, transcription factors, Ca^2+^ channels, ion transporters, and proinflammatory genes.^52^ In diabetes and hypertension, oxidative stress (increased ROS bioavailability) promotes vascular inflammation, fibrosis, and injury, processes that are normalized by Nox inhibitors or ROS scavengers, or both. Nox1, but not Nox4, seems to be important in atherosclerosis in diabetes, as we demonstrated in Nox1-deficient mice on the atherosclerosis-prone ApoE^−/−^ background made diabetic with streptozotocin.^53^ Nox4 has been implicated in renal injury in mouse models of diabetes, effects that are ameliorated with Nox1/4 inhibitors and in mice deficient in Nox4.^54^, ^55^ Nox5 may also be important in diabetes-associated vascular injury and nephropathy. We demonstrated that renal Nox5 expression is increased in patients with diabetic nephropathy. Moreover, in transgenic mice with podocyte-specific expression of human Nox5, renal injury was amplified by diabetes.^56^ Similar findings were observed in mice expressing human Nox5 in a vascular smooth muscle cell-specific manner.^57^ Although extensive experimental evidence showed a renoprotective effect of Nox4 inhibition in diabetes, a recent clinical study using GKT137831, a Nox1/4 inhibitor, failed to show improvement in renal function in patients with diabetic nephropathy.^58^ Whether targeting Nox5 may have better clinical outcomes is unclear, because to date there are no Nox5 inhibitors available.

### Inflammation and the immune system

Links between inflammation and the immune system with metabolic dysfunction, hypertension, and cardiovascular morbidity are supported by extensive experimental data.^59^ This encompasses a number of immune metabolic aspects, including the key role of the tricarboxylic cycle or sphingosine-1-phosphate in the regulation of vascular inflammation.^59^, ^60^ Clinical studies have shown that patients with T2D have increased total leukocyte counts, particularly neutrophils and lymphocytes, that correlate with insulin sensitivity,^61^ which is in part mediated by inflammatory changes of adipose tissue.^62^ Inflammatory biomarkers are also useful in developing targeted cardiovascular therapies in the context of metabolic dysfunction.^63^ The link between inflammation and T2D is further supported by genetic studies and clinical trials showing protective effects of immune-targeted therapies and anti-inflammatory actions of classic antidiabetes drugs.^64^ Circulating and locally produced effector cytokines such as TNF-α, interferon-γ, IL-1β, and IL-12 may influence insulin sensitivity of peripheral tissues and can modulate insulin release in the pancreatic islets.^65^, ^66^, ^67^, ^68^ Increased glucotoxicity and lipotoxicity have been associated with immune cell infiltration of target tissues, thereby affecting diabetes-associated target organ damage and cardiovascular complications,^68^, ^69^ including the development of metabolic cardiomyopathy.^70^, ^71^ Inflammation is a key modulator of metabolic and diabetic CVD.

#### Genetic evidence

Although genome-wide association studies (GWAS) for insulin resistance or T2D have not shown strong associations with immune-related genes, numerous metabolic traits are linked to immune-related loci.^72^ Studies integrating metabochip approaches with GWAS have shown that classic immunometabolic genes including JNK signalling pathways (such as *MAP3K1*), nuclear factor kappa B (NF-κB) regulators (*MACROD1*), inflammasome activators (NRF3), and interferon-γ receptor genes associate with T2D.^73^, ^74^ This also corresponds to results of recent large T2D GWAS that identified genes related to macrophage function and antigen presentation (*MAEA*, *ST6GAL1*), and T-cell signalling (*CMIP* or *PTPRJ*).^72^, ^75^ While trying to interpret these important studies, it should be appreciated that GWAS approaches have limitations, because only a small component of heritability of complex traits is directly explainable by single-gene variability.^76^

#### Clinical evidence

Increasing clinical evidence indicates an immune component in T2D and its cardiovascular complications. Immune-targeted therapies currently available for the treatment of rheumatoid arthritis and autoimmune disorders, including anti-TNF therapies, may prevent insulin resistance as well as cardiovascular risk.^64^, ^77^ A recent meta-analysis of studies with anti-TNF agents supports an overall protective effect of anti-TNF therapies on lifetime risk of diabetes as well as insulin sensitivity and obesity.^74^

A recent large proof of concept trial of anti-inflammatory therapy in patients after myocardial infarction (A Randomized, Double-blind, Placebo-controlled, Event-driven Trial of Quarterly Subcutaneous Canakinumab in the Prevention of Recurrent Cardiovascular Events Among Stable Post-Myocardial Infarction Patients With Elevated hsCRP [CANTOS]; canakinumab targeting IL-1β) showed a clear reduction in the rate of cardiovascular events, albeit with an associated increase in the rate of severe infections.^78^ These results were particularly evident in high-risk patients, although effects on metabolic profile remain unclear.^78^ However, evidence that IL-1β targeting may have significant metabolic benefits has been well established, as evidenced by improved profile insulin sensitivity in response to IL1-β blockade.^79^ The potential beneficial effects of anti-inflammatory and immune-modulating agents in T2D and its complications may relate to direct vasoprotective effects. These studies have led to the rapid development of the concept of immunometabolism, clearly linking metabolic changes in the tissues to the regulation of inflammation as well as metabolic status of immune cells to their activation.^28^, ^56^ The latter can be characterized by a switch between oxidative phosphorylation and anaerobic glycolysis, which is observed in macrophages and T cells.^30^, ^59^ This also emphasizes the importance of the interplay between vascular oxidative stress and the development of inflammation in adipose tissue and the vasculature.

#### Anti-inflammatory properties of antidiabetic therapies

Classic approaches improving metabolic health, such as weight reduction and the use of metformin, statin drugs, pioglitazone, and insulin have been shown to have anti-inflammatory effects. Metformin reduces C-reactive protein levels by 13%. More recently, a novel anti-inflammatory mechanism of metformin affecting M1/M2 polarization of macrophages has been shown to reduce obesity-associated low-grade inflammation, possibly because of adenosine monophosphate-activated protein kinase (AMPK) activation. These effects were modulated by AMPK and the AMPK analogue 5-aminoimidazole-4-carboxamide ribonucleotide, effects that appear stronger than those of metformin.^80^ Recent studies have shown that salicylates have anti-inflammatory effects that involve inhibition of NF-κB and that they also prevent diabetes and improve insulin resistance in experimental models and humans.^81^, ^82^ Drugs such as glicazide and troglitazone, as well as N-acetylcysteine, decrease inflammatory markers in patients with diabetic nephropathy and diabetic retinopathy.^83^

Epigenetics is another mechanism that may influence inflammation and immunometabolism in diabetes.^59^ Histone deacetylase (HDAC) inhibitors cause NF-κB inhibition through acetylation of the p65 subunit. Givinostat (formerly ITF2357), an orally active HDAC inhibitor, has been shown to prevent the development of diabetes.^84^, ^85^ Similarly, activation of sirtuin1, which is involved in inflammation, metabolism, and aging, has been shown to have anti-inflammatory properties in diabetes.^86^

## MiRNAs, Diabetes, and Vascular Complications

miRNAs are a group of noncoding RNAs that are multifunctional. They fine tune gene expression and have been implicated in various pathologic processes, including T2D and the development of diabetic vascular complications. A number of pancreatic B-cell–specific miRNAs have been identified, including miR-375, miR-124a, miR-96, miR-7a, miR7a2, miR-30d, miR-9, miR-200, miR-184, and let-7.^87^ These play a role in pancreatic function, insulin secretion, and glucose tolerance. Differential miRNA signatures have been identified among prediabetic individuals, patients with diabetes, and patients with diabetes and vascular complications, suggesting that miRNAs may be novel biomarkers. Diabetic cardiovascular complications are associated with increased levels of miR-223, miR-320, miR-501, miR504, and miR1 and decreased levels of miR-16, miR-133, miR-492, and miR-373.^9^ Whether these changes in miRNA are simply biomarkers of disease or whether they are directly involved in the vasculopathy of diabetes remains unclear.

### Treatment of diabetes mellitus and its cardiovascular complications

Once T2D has been diagnosed, the aim of achieving glucose control is principally to avoid microvascular complications. There are some benefits with respect to macrovascular complications, but this is dependent on the profile of individual drug classes and even appears to be different for agents within the same class.^88^ The role of BP lowering to improve prognosis in T2D has been established since the **UK P**rospective **D**iabetes **S**tudy (UKPDS) in 1998.^89^, ^90^ However, more recently, more widespread use of glucose-lowering agents that reduce (rather than increase) weight, lower BP, and have beneficial “off-target” effects (as demonstrated in recent large cardiovascular outcome trials) facilitates cardiovascular risk factor control and is playing a role in improving the cardiovascular prognosis of T2D.^91^, ^92^

Achieving glucose control in T2D begins with weight management. Particularly in the first 8 years after diagnosis, normal glucose tolerance can be restored if radical weight reduction can be achieved, most effectively using a very low calorie liquid replacement diet.^93^ In obese patients, this can also occur after successful bariatric surgery, particularly the Roux-en-Y procedure.^94^ The mechanism may involve reduction in ectopic fat, and consequent relief from its proinflammatory effects, in and around the pancreatic islets of Langerhans.^95^

All current glucose-lowering guidelines suggest the early addition of metformin as first-line therapy. Unlike the sulphonylureas, which augment insulin secretion, metformin lowers blood glucose levels principally by decreasing hepatic glucose production and promoting weight reduction (with little effect on BP). Among the many proposed mechanisms of action of metformin is activation of AMPK: This is now thought to be a secondary effect of inhibition of the mitochondrial respiratory chain.^96^ Such effects of metformin may act directly (ie, independent of blood glucose lowering) on other tissues, including vascular endothelial cells. Metformin treatment is associated with improvements in endothelial biomarkers and reduction in plasma high-sensitivity C-reactive protein levels.^97^, ^98^ It was associated with cardiovascular benefit in the landmark UKPDS.^99^

Other second-line agents used in glucose lowering include pioglitazone, a thiazolidinedione that directly promotes the differentiation of adipocytes within subcutaneous adipose depots (by activation of peroxisome proliferator-activated receptor-γ), thus promoting storage of non-esterified fatty acids (NEFAs).^100^ Pioglitazone reverses many of the metabolic features associated with insulin resistance without much effect on BP. Anti-inflammatory effects have been demonstrated in human adipose tissue biopsy samples and also in some animal models.^101^ There was great hope in the 1990s that agents from this class would have major benefits for the cardiovascular system, a hypothesis that was to some extent supported by the results of the **Pro**spective Pioglit**a**zone **C**linical Trial in Macro**v**ascular **E**vents (PROACTIVE) cardiovascular outcome trial, although beneficial effects were offset by weight gain and fluid retention.^102^

More recently introduced classes of glucose-lowering agents have heralded an exciting era in T2D pharmacotherapy because they are associated with weight reduction, BP reduction, and, importantly, reduced rates of major adverse events in long-term cardiovascular outcome trials.^91^, ^92^, ^103^

Glucagon-like peptide-1 agonists are injectable agents that augment glucose-dependent insulin secretion (the “incretin” effect), delay gastric emptying (enhancing satiety), and have central effects on hypothalamic nuclei to reduce appetite.^104^ Systolic BP is lowered beyond the effect that would be expected purely from weight loss, and there is an improvement in pulse-wave velocity, reflecting a reduction in arterial stiffness. However, the time course of cardiovascular event reduction in the **L**iraglutide **E**ffect and **A**ction in **D**iabetes: **E**valuation of Cardiovascular Outcome **R**esults (LEADER) trial suggests a primary antiatherosclerotic rather than hemodynamic effect.^91^ Indeed, liraglutide has been shown to have anti-inflammatory actions on the cardiovascular system in a number of preclinical and clinical studies.^103^, ^105^

SGLT2 inhibitors promote lowering of the threshold for urinary glucose excretion: an additional glucose equivalent to 300 kcal per day is therefore cleared by the kidneys, promoting weight loss and a catabolic state with increased circulating ketone bodies and NEFAs.^106^ There are associated reductions in BP and plasma volume, which together may have been responsible for the early reduction in cardiovascular event rates seen with empagliflozin in Empagliflozin, Cardiovascular Outcomes, and Mortality in Type 2 Diabetes (EMPA-REG OUTCOME) trial.^91^ A shift in fuel substrate metabolism from glucose to NEFAs and ketones, including by the myocardium, is 1 of the mechanisms by which SGLT2 inhibitors may provide cardiovascular protection,^107^ but studies in apoE knockout mice and Zucker diabetic fatty rats suggest that anti-inflammatory effects may also play a role.^108^

### Diabetes, vasoprotection, and potential new therapies

Data from landmark clinical trials in T2D including UKPDS, ADVANCE, and **A**ction to **C**ontrol **C**ardi**o**vascular **R**isk in **D**iabetes (ACCORD) demonstrate that treating comorbidities including hypertension and hypercholesterolemia is a more effective strategy for reducing cardiovascular complications than targeting blood glucose levels with conventional agents.^109^ Antihypertensive drugs such as angiotensin-converting enzyme inhibitors, angiotensin-receptor blockers, mineralocorticoid-receptor blockers, and calcium-channel blockers may have direct vasoprotective effects, and their use may contribute, at least in part, to reduced vascular complications in patients with diabetes and concomitant hypertension.^110^ Tight control of BP has been shown to reduce cardiovascular risk in T2D: most recent US and Canadian guidelines recommend a target of < 130/80 mm Hg.^111^, ^112^ Statin drugs and clopidrogel are also vasoprotective and may have extra benefit in patients with diabetes. Some of the beneficial effects of these drugs have been attributed to their antioxidant and anti-inflammatory properties.

New therapeutic approaches targeting oxidative stress, inflammation, and fibrosis are currently being developed to treat diabetes-associated cardiovascular complications.^113^ In particular, drugs that increase Nrf-2 activity, such as bardoxolone methyl, and strategies to inhibit the pyrin domain containing 3 (NLRP3) inflammasome, may have therapeutic potential. A novel bardoxolone methyl derivative, dh404, has been shown to attenuate endothelial dysfunction, reduce Nox1 expression, decrease oxidative stress, and inhibit inflammation in diabetic mice, suggesting that upregulation of Nrf2 may have therapeutic potential to limit diabetes-associated vascular damage.^114^ Another example includes inhibition of dipeptidyl peptidase-4 by linagliptin, which reduces obesity-related insulin resistance and inflammation by regulating M1/M2 macrophage status.^115^ Other therapies on the horizon for the treatment of cardiovascular complications of diabetes include pentoxifylline (methylxanthine derivative and nonspecific phosphodiesterase inhibitor with anti-inflammatory and antifibrotic effects), ruboxistaurin (selective protein kinase C-β inhibitor), pirfenidone (TGF-β inhibitor), bindarit (MCP-1/CCL2 inhibitor), sulodexide (an oral formulation composed of 2 glycosaminoglycans), AKB-9778 (Tie2 activator), baricitinib (JAK/STAT inhibitor), and Nox inhibitors.^116^ The clinical benefit of these compounds awaits further confirmation, and novel nanotherapeutic approaches are being developed to target inflammation.^117^

## Conclusions

Diabetes is associated with an increased risk of CVD, which is exaggerated with coexistent hypertension. Many of the underlying molecular mechanisms, including oxidative stress, inflammation, and fibrosis causing microvascular and macrovascular complications of diabetes, also cause vascular remodelling and dysfunction in hypertension. Controlling comorbidities, especially hypertension, and targeting strategies to promote vascular health, may be especially important in reducing the microvascular and macrovascular complications of diabetes.

## Funding Sources

This work was supported by grants from the British Heart Foundation (RG/13/7/30099, RE/13/5/30177), the Wellcome Trust Senior Biomedical Fellowship (to T.J.G.), and the National Science Centre of Poland (2011/03/B/NZ4/02454).

## Disclosures

The authors have no conflicts of interest to disclose.
